# Group based prenatal care in a low-and high risk population in the Netherlands: a study protocol for a stepped wedge cluster randomized controlled trial

**DOI:** 10.1186/s12884-016-1152-0

**Published:** 2016-11-15

**Authors:** Birgit S. van Zwicht, Matty R. Crone, Jan M. M. van Lith, Marlies E. B. Rijnders

**Affiliations:** 1 Department of Obstetrics, Leiden University Medical Center, PO Box 9600, 2300 RC Leiden, The Netherlands; 2 Department of Public Health and Primary Care, PO Box 9600, 2300 RC Leiden, The Netherlands; 3 Department of Child Health TNO, PO Box 2215, 2301 CE Leiden, The Netherlands

**Keywords:** Group Prenatal Care, Group Care, Pregnancy Outcome, Health Behavior, Patient Satisfaction, Prenatal care/methods, Infant, Newborn, Group Processes, Patient Education, CenteringPregnancy

## Abstract

**Background:**

CenteringPregnancy (CP) is a multifaceted group based care-model integrated in routine prenatal care, combining health assessment, education, and support. CP has shown some positive results on perinatal outcomes. However, the effects are less obvious when limited to the results of randomized controlled trials: as there are few trials and there is a variation in reported outcomes. Furthermore, former research was mostly conducted in the United States of America and in specific (often high risk) populations. Our study aims to evaluate the effects of CP in the Netherlands in a general population of pregnant women (low and high risk). Furthermore we aim to explore the mechanisms leading to the eventual effects by measuring potential mediating factors.

**Design:**

We will perform a stepped wedge cluster randomized controlled trial, in a Western region in the Netherlands. Inclusion criteria are <24 weeks of gestation and able to communicate in Dutch (with assistance). Women in the control period will receive individual care, women in the intervention period (starting at the randomized time-point) will be offered the choice between individual care or CP. Primary outcomes are maternal and neonatal morbidity, retrieved from a national routine database. Secondary outcomes are health behavior, psychosocial outcomes, satisfaction, health care utilization and process outcomes, collected through self-administered questionnaires, group-evaluations and individual interviews. We will conduct intention-to-treat analyses. Also a per protocol analysis will be performed comparing the three subgroups: control group, CP-participants and non-CP-participants, using multilevel techniques to account for clustering effects.

**Discussion:**

This study contributes to the evidence regarding the effect of CP and gives a first indication of the effect and implementation of CP in both low and high-risk pregnancies in a high-income Western society other than the USA. Also, measuring factors that are hypothesized to mediate the effect of CP will enable to explain the mechanisms that lead to effects on maternal and neonatal outcomes.

**Trial registration:**

Dutch Trial Register, NTR4178, registered September 17^th^ 2013.

## Background

In the Netherlands, perinatal mortality and morbidity is relatively high compared to other European countries. One in six children is born with health problems, defined as congenital anomalies, preterm birth, low birth weight and a low Apgar score [1]. Risk factors for adverse perinatal outcomes are amongst others life style (e.g. in the Netherlands 11% of the women with a lower education level smoke during pregnancy [2]), psychological issues [3], work related risks [4], medication use [5] and chronic illnesses [6]. In addition, perinatal outcomes are worse for ethnic minorities (non-Western ethnicity) [7, 8] and for Western women living in deprived urban areas [9, 10]. Influencing these risk factors could lower the perinatal morbidity rate. Often, however, interventions targeted at the general population do not reach (ethnic) minorities and have limited effects on health behavior [11–13].

In 2012, CenteringPregnancy (CP) was introduced in the Netherlands [14]. CP, developed in the United States of America (USA), is a model of prenatal group based care, within which one-to-one visits are being replaced by group consultations [15]. CP combines the three major components of care – health assessment, education, and support. The group based character of CP allows more time for self-management, education, skill building, and caregiver-patient interaction [16–18]. In addition, the group process can strengthen patient’s self-efficacy by vicarious learning and modeling by seeing others successfully overcoming barriers and accomplishing desired behavioral changes [19–22]. Next, CP is integrated in the Dutch routine prenatal care, which is freely accessible to all women in the Netherlands (in 2014 173,544 women received prenatal care [23]). The integrated character improves the reach of the preventive activities that are embedded in CP, as was shown by a former Dutch study concluding that women from an ethnic minority group evaluated a prevention program that was fully integrated in routine midwifery care as highly acceptable and satisfactory [24]. In summary, CP has the potential to effectively influence the risk factors for adverse perinatal outcomes to reach high-risk groups.

Research on CP has shown some positive effects: amongst others on health literacy [25, 26], preparedness for labor and birth [17], and initiating breastfeeding [17, 27]. Women reported receiving more social support [28, 29], and were more satisfied with provided care [17, 28–30]. Moreover, the number of preterm births significantly decreased in a high-risk population, the mean birth weight increased and the number of women who received substandard care was reduced [17, 29].

However, a part of the evidence is derived from non-randomized studies. The evidence from randomized controlled trials (RCT) is less obvious, which is due to the limited number of RCT’s on prenatal group-based care and the variation in the reported outcomes [17, 27, 30, 31]. Recently, Caitlin et al. concluded that additional research is required to assess the effects of prenatal group-based care models, such as CP, on neonatal and maternal outcomes [26]. Furthermore, most results of the effect of CP originate from experimental studies conducted in high-risk populations and in the USA [17, 29, 31–34]. It is unknown what the effect of CP is in a different maternity healthcare system and in the general population.

The aim of the present study is to evaluate the effects of CP in the Netherlands in primary health care (low risk population) as well as in hospital based care (moderate to high risk population) on neonatal and maternal health outcomes, health behaviors and psychosocial outcomes. The effectiveness of CP will be established by comparing the outcomes in the intervention group (prenatal care within the CP model) and the control group (usual individual care). Primary hypotheses are that in comparison to the control group, the intervention condition will lead to better neonatal and maternal outcomes. Secondary hypotheses are that the effect on the outcome variables is mediated by (changes in) health literacy, self-regulation, health behavior, health care utilization as well as level of group cohesion and level of implementation.

## Methods

### Design

This study will be performed in thirteen midwifery practices (primary care, low-risk population) and two hospitals (secondary care, moderate to high-risk population) that do not yet offer CenteringPregnancy within the region ‘northern South-Holland’ (an urban – sub-urban setting). A stepped wedge cluster randomized controlled trial design is chosen for several reasons. Firstly, randomization at individual level is not appropriate, as training professionals in CP will most probably also affect the non-CP consultations. Secondly, since midwifery care in the Netherlands is subjected to marketing forces, cluster randomization is expected to result in unfair competition between independent centers operating in the same region. Within the chosen design, all clusters (midwifery practice or hospital) start to collect control data at the same time (control period). Clusters will subsequently implement the intervention at a different, randomly selected time point and will then start to collect intervention data (intervention period). For this study, we will divide all fifteen centers into three groups and will randomly assign each group to one of three time points (steps) at which they will start to implement the intervention, with a between-step period of 3 months.

### Data collection

All women that register for prenatal care at a participating center will receive written information about the study. All women under 24 weeks of gestational age (GA) at inclusion who are able to communicate in Dutch (with assistance) will be asked to participate in the study. Their care provider will verbally inform the women at their first prenatal consultation (generally around 8–12 weeks of GA) and will ask them for informed consent. No exclusion criteria will be employed. Participation includes permission to collect women’s routine pregnancy outcomes as registered in the National Dutch Perinatal Data Registry (Perined, see end of this section) and optional agreement to fill out four self-administered questionnaires. For participants under the age of 18, informed consent of their parents or caregivers will be obtained.

#### Control group

**Fig. 1:**
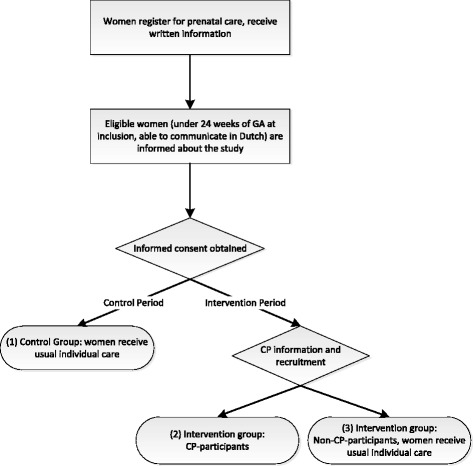
Flow-chart data collection

Within the control period, all participants will be part of the control group and will receive usual individual prenatal care. See Fig. 1.

#### Intervention group

Within the intervention period, pregnant women are offered perinatal care in the CP model. In the CP model traditional prenatal care with one-to-one visits with a perinatal care provider is replaced by the use of a group model [35]. Care is provided by a midwife or an obstetrician (facilitator) and a co-facilitator to groups of eight to twelve women of similar gestational age. Groups meet eight to ten times during pregnancy at the usual scheduled visits, with sessions running for 90 to 120 min. The usual prenatal health assessment is integrated with information, education and peer support. All involved care providers will be trained by licensed trainers to perform CP. The intervention period begins when the center starts to recruit for their first CP group, after the randomized time point. All women included in the study during this period will be part of the intervention group. In addition to the study information, participants will be informed about CP at their first prenatal consultation and will be given the choice to participate in CP. Women that do not participate in CP will receive usual individual care, resulting in two sub-groups within the intervention group. See Fig. 1.

We will use data from the Perined database. The Perined database contains linked and validated routine care information concerning pregnancy, delivery, (re)admissions and pregnancy outcomes [36]. Data are routinely and separately registered by midwives, obstetricians, general practitioners and paediatricians/neonatologists. For this study, the Perined data will be complemented with data from the questionnaires to be completed by participating women at four time points: Table 1 provides an overview of the timing of the different items. Reminders will be sent after 1, 2 and 3 weeks and participants will be reminded by their care provider. Finally, we will use implementation data from CP-group-evaluations and qualitative data from semi-structured interviews with care providers. Outcome measurements are described below. More details on instruments used in the questionnaires are provided in Table 2.

**Table 1:** Overview of items in the questionnaires at four measurement time points

T1, inclusion (8–12 weeks of GA)	T2 (28 weeks of GA)	T3 (36 weeks of GA)	T4 (6 weeks postpartum)
Basic characteristics, current weight	Life style Knowledge (2)	Life style (3), medication use (2)	Life style (4), prenatal and perinatal health care use (2)
Life style and medication use	Life style (2)	Prenatal health care use	Labor experience, and emotions during labor
Preconceptional health care use	Depression	Prenatal Care Knowledge (2)	Breastfeeding initiation and duration
Life style- and Prenatal care Knowledge	Participation and experience of CP^a^	Stress and Coping (2)	Neonatal sleeping- and crying behavior
Stress and Coping		Social Support (2)	Self-efficacy in child care
Social Support		Readiness for Labor and Baby care	Depression (2)
Intended pregnancy education		Expectations on labor and child birth, intentions on infant feeding	Pregnancy education (2)
Intended participation CP^a^		Prenatal Care Satisfaction	Participation CP (3)^a^
		Participation and experience of CP(2)^a^	

^a^Complementary items for intervention group

**Table 2:** Overview of instruments that will be used in the study

Tool	Constructs	Items	Scale — Analysis	Validation	References
Labor and birth outcomes					
Readiness for labor-item	On a scale of 0 to 100, where 0 is not at all and 100 is completely, how ready do you feel for labor and delivery?	1 item	0-100 – total score	Not available	Ickovics et al., 2007 [17]
Labor and Delivery Index (LADY-X)	Quality of received care and maternal emotions	7 items: e.g. “Information given by the healthcare professionals during childbirth.”	3 point scale^a^ – sum score (range 0–14)	Test-retest reliability, ICC^b^ > .80; Convergent and divergent validity *r* = .24-.61; Construct validity *p* < .001-.02.	Gärtner et al., 2015 [37]
Shortened Labor Agentry Scale (LAS-10)	Sense of control during childbirth	10 items: e.g.”I felt confident.”	7 point scale (almost always-rarely) – sum score (range 10–70)	A^c^ = .85-.97^d^	Hodnett and Simons-Tropea, 1987 [38] Geerts et al., 2014 [59]
Health literacy					
Prenatal/Postnatal care knowledge	Prenatal and postnatal care knowledge	19 items: e.g.” Babies of mothers who smoke tend to be smaller than babies of mothers who do not smoke.”	5 point scale (definitely false-definitely true) – sum score (range 0–95)	α = .65	Ickovics et al., 2007 [17]
Psychosocial outcomes					
Revised Prenatal Distress Questionnaire (NuPDQ)	Concerns about birth/baby, concerns about weight/body image, concerns about emotions/relations	9-17^e^ items: e.g. “Are you feeling bothered, worried or upset at this point in your pregnancy about taking care of a new born baby?”	3 point scale (not at all-very much) – sum score (range 0–34)	Test-retest reliability *r* = .75, α = .80-.81^d^	Yali and Lobel, 1999 [39] Yali and Lobel, 2002 [40] Alderlice et al., 2012 [41]
Cambridge Worry Scale (CWS)	Women’s major worries in pregnancy: socio-medical, health, socio-economic, and relational	17 items: e.g. “How much of a worry is your housing to you?”	6 point scale (not a worry-major worry) – at item level or using total (range 0–85) or factor scores	*Socio-medical* α = .71; *Health* α = .70; *Socio-economic* α = .29-.63; *Relational* α = .67. Test-retest reliability *r* = .69-.72	Green and Kafetsios, 1997 [42] Green et al., 2003 [47]
Coping (based on the Revised Prenatal Coping Inventory)	Problem focused active coping, emotion focused active coping, emotional passive coping	9 items: e.g. “How often did you try not to think about it?”	5 point scale (never-very often) – sum score per subscale (range 0–12)	Not available	De Ridder et al., 1996 [48] De Ridder et al., 1998 [49] Savelkoul et al., 2000 [50] Hamilton and Lobel., 2008 [51]
Social Support List-12 Interaction (SSL-12 I)	Daily support, problem support and appreciation support	12 items: e.g. “Do you get invited to a party or dinner sometimes?”	4 point scale (seldom-very often) – scale scores (range 4–16) and sum score (range 12–48)	*Daily support* α = .70-.80^d^; *Problem support* α = .72-.89^d^; *Appreciation support* α = .72-.82^d^.	Kempen et al., 1995 [60] Bridges et al., 2002 [53] Van Sonderen., 2012 [52]
Edinburgh Postnatal Depression Scale (EPDS)	Prenatal and postnatal depression The EPDS was originally developed for postnatal use, but was validated as a prenatal screening instrument.	10 items: e.g. “I have been able to laugh and see the funny side of things.”	4 point scale^a^ – sum score (range 0–30)	α = .80	Cox et al., 1987 [54] Murray et al., 1990 [61] Green et al., 1994 [62] Adouard et al., 2004 [63]
Parenting outcomes					
Readiness for baby care-item	“On a scale of 0 to 100, where 0 is not at all and 100 is completely, how ready do you feel for taking care of your baby?”	1 item	0-100 – total score	Not available	Ickovics et al., 2007 [17]
Parental Expectations Survey (PES)	Women’s self-efficacy in child care	25 items: e.g. “I can manage the feeding of my baby.”	10 point scale (cannot do-certain can do) – sum score (range 25–250)	α = .86-.91^d^	Reece et al., 1992 [55] Reece et al., 1998 [64] McCarter-Spaulding et al., 2001 [65]
Satisfaction with prenatal care					
Patient Participation and Satisfaction Questionnaire (PPSQ)	Participation in prenatal care and satisfaction	22 items: e.g. “Helpful information was given to me about my pregnancy.”	5 point scale (not applicable and very dissatisfied-very satisfied) – scale scores and total sum score (range 22–110)	*Participation* α = .93; *Satisfaction* α = .93; *Total* α = .95.	Littlefield et al., 1987 [56] Ickovics et al., 2007 [17]

^a^Verbal aspects vary per item

^b^Intraclass correlation coefficient

^c^Cronbach’s alpha

^d^Varying between studies

^e^Number of items vary depending on time point administered

### Outcomes

#### Basic characteristics

The basic characteristics that will be collected are age, (parental) country of birth, religion, educational level, marital status, employment status, and parity.

#### Neonatal health outcomes

The primary neonatal outcomes are perinatal mortality (defined as death per 1000 still- and life births from a GA above 22 weeks to 7 days postpartum) and perinatal morbidity. Perinatal morbidity (composite outcome) is defined as presence of congenital abnormalities, small for gestational age (birth weight below 10^th^ percentile), preterm birth (<37 weeks of GA), Apgar score after 5 min < 7 and/or admission to a neonatal intensive-care unit (immediately after birth). These outcomes will be retrieved from the Perined database, as well as gestational age at birth and birth weight. Other neonatal outcomes that will be addressed in the self-administered questionnaires are child’s crying- and sleeping behavior per 24 h (and to what extent women’s expectations on crying behavior are met), consultations with a general practitioner and/or pediatrician and admission to the hospital within 6 weeks postpartum.

#### Maternal health outcomes

We will collect data on maternal mortality and morbidity (defined as intensive care admission, eclampsia/HELLP, and/or postpartum hemorrhage ≥ 1000 ml), and prenatal and perinatal referrals to specialized obstetrical care.

#### Labor and birth outcomes

Birth outcomes that will be collected are: intended and actual place of delivery, type of delivery (spontaneous or induced), mode of delivery (non-operative vaginal delivery, operative vaginal delivery, and planned and unplanned cesarean section), augmentation (yes/no), and need for analgesia (epidural, remifentanil, pethidine). These outcomes will be retrieved from the Perined database and complemented by data from the questionnaires regarding expectations on labor pain, intentional use of analgesia, perceived duration of delivery (Table 1). We will measure women’s readiness for labor using an item designed by Ickovics et al. [17]. Women’s delivery experience will be measured with the Labor and Delivery Index (LADY-X) [37] and women’s sense of control during delivery with the Labour Agentry Scale (LAS-10) [38].

#### Health behavior outcomes and health literacy

Health behavior outcomes and health literacy will be collected using the questionnaires. Health behavior outcomes are: physical activity (number of 30-min exercise per week and self-judgment physical activity), nutritious behavior (breakfast-, vegetable-, fruit-, juice- and snack-consumption, and self-judgment eating behavior), substance use (tobacco-, alcohol-, and soft or hard drug consumption, and intentions towards smoking cessation), medication use (folic acid use and use of (prescribed) drugs), intention of infant feeding and attitude towards breastfeeding, and infant feeding on day one, at 1 week postpartum and at 6 weeks postpartum. Women’s prenatal and postnatal care knowledge will be measured with a scale developed by Ickovics et al. [17].

#### Psychosocial outcomes

Psychosocial outcomes include perceived stress, coping, social support, and depression. Stress will be measured using the Prenatal Distress Questionnaire (NuPDQ), which is a revised version of the original 12-item scale developed by Yali and Lobel in 1999 [39–41]. Socio-economic and relational stress will be measured by four items of the Cambridge Worry Scale (CWS), complementing the NuPDQ [42–47]. We have added a concluding item on the amount of experienced stress on a scale from 0.0 to 10.0. To measure coping we will use a self-developed short instrument measuring three constructs based on the coping strategies as described by De Ridder & Schreurs (1994), and Savelkoul et al. (2000): problem focused active coping, emotion focused active coping and emotional passive coping [48–50]. Each coping strategy is measured on a subscale and contains three items, which are based on the items of the revised Prenatal Coping Inventory (NuPCI), using the same answering scale (5 point Likert like scale, 0 = never tot 4 = very often) [51]. For example: “How often did you take a walk or performed other physical exercise to feel better?” (emotion focused active coping), “How often did you try not to think about it?” (emotional passive coping), and “How often did you talk to others in the same situation?” (problem focused active coping). Social support will be measured using the social support list-12 Interaction [52, 53]. Depression will be measured using the Edinburgh Postnatal Depression Scale (EPDS) [54].

#### Parenting outcomes

We will measure women’s readiness for baby care using an item designed by Ickovics et al. [17]. Women’s self-efficacy in child care will be evaluated using the Parental Expectations Survey (PES) [55].

#### Satisfaction with prenatal care

Women’s experience and satisfaction with prenatal care will be evaluated using the Patient Participation and Satisfaction Questionnaire (PPSQ) [56, 57].

#### Process outcomes

We will assess the implementation of CP by monitoring the percentage of women that start CP, the addressed content within the group sessions, the involvement of women and their partners (adherence), and model fidelity. These data will be collected from the CP-group-evaluations, filled out by the group facilitators at the end of each session. These evaluations also contain data on group cohesion. Women’s experience with CP and their inhibiting and facilitating factors to participate will be addressed in the self-administered questionnaires: see Table 1. Inhibiting and facilitating factors in the implementation process and the care providers satisfaction with prenatal care will be addressed in individual semi-structured in-depth interviews. Health care utilization of women will be measured in the self-administered questionnaires, addressing preconceptional and prenatal utilization of general health care, and health care utilization provided by a perinatal care center.

### Statistical issues

#### Sample size calculation

A minimal sample of 600 pregnant women in both the intervention and control condition is needed to be able to accept with 95% confidence and an upper confidence limit less than 1.85, that after the intervention has been implemented, there is at least no significant difference in infant morbidity (using 14% of infant morbidity as outcome) between CP and individual care. This sample size is also largely sufficient to find amongst others a difference in prenatal care satisfaction or in proportion of breastfeeding comparable to Ickovics et al, with an α of 0.05 and a power of 0.90 [17]. We will account for a 20% loss to follow-up and aim to include in total 1600 women (800 in both conditions).

#### Data analysis

Data entry will be automatic by using a secured online survey system or (in case of hard copy) scanned and checked using the Teleform software. Data will be stored in a digital data base only accessible to the researchers. Before analysis, all data will be cleaned improving data quality.

We will conduct intention-to-treat analyses to compare the primary and secondary outcomes of the control group with those of the complete intervention group. Also a per protocol analysis will be performed comparing the outcomes of the three subgroups (see Fig. 1): control group (1), CP-participants (2) and non-CP-participants (3). Analyses will be descriptive and inferential (univariate and multivariate), and we plan to perform multilevel techniques to account for the clustering effect among participants in centers and CP-participants in CP-groups. The analysis of the implementation data and health care use will be descriptive. The relation between the degree of implementation and primary and secondary outcomes will be analyzed using multi-level analysis. Furthermore, the interviews with care providers will be conducted using a topic list based on the results of the analysis of the implementation data. They will be transcribed and qualitatively analyzed using a framework approach.

## Discussion

Former research has shown a positive effect of CP, including a higher birth weight and more prenatal care satisfaction, however the findings were inconsistent [17, 26, 27, 30, 31]. Furthermore, due to differences in population characteristics and health care system, previous findings cannot be directly extrapolated to the Dutch setting. Our study aims to contribute to the evidence regarding the effect of CP and will give a first indication of the effect and implementation of CP in a Western high-income society other than the USA. Also, by measuring factors that are hypothesized to mediate the effect of CP, we aim to be able to explain the mechanisms that lead to eventual found effects on maternal and neonatal outcomes.

A strength of our study lies in the stepped wedge cluster design. In this design, participating practices are randomly assigned to the period in which they will start providing CP and will function as their own control group before implementing CP. Also, it allows to take into account time effects. Another strength of the study is the use of routine data to assess several neonatal and maternal outcome measures.

One limitation however is the fact that centers are not yet trained in CP and that it takes some time to implement, which may lead to less model fidelity and smaller effect sizes. Fidelity to facilitate group processes in CP and content fidelity are known to associate with significant reductions in preterm birth and intensive utilization of care [58]. To gain more insight in the effects of fidelity of the model as provided by health care providers we will collect data on process and implementation, and will relate it to the effects of CP. The possibility of selection bias by letting women choose between CP or individual care is another limitation of our study. To be able to estimate the degree of selection bias, we will collect numerous demographic and psychosocial data of both groups in the intervention period (CP-participants and Non-CP-participants), also allowing us to correct for differences in basic characteristics. In addition, we will perform an intention-to-treat analysis, as well as a per protocol analysis. Differences in results of both analyses will provide an indication of assumed selection bias.

Abovementioned limitations of the study are at the same time the strengths of our study, since our chosen design and methods are pragmatic, allowing us to evaluate the effect of CP when implemented in daily practice.

## Abbreviations

(Nu)PDQ: Prenatal Distress Questionnaire
CP: CenteringPregnancy
CWS: Cambridge Worry Scale
EPDS: Edinburgh Postnatal Depression Scale
GA: Gestational Age
LADY-X: Labor and Delivery Index
LAS: Labour Agentry Scale
NuPCI: Prenatal Coping Inventory
PES: Parental Expectations Survey
PPSQ: Patient Participation and Satisfaction Questionnaire
RCT: Randomized Controlled Trial
SCL-90: Sympton Checklist-90
SSL-I 12: support list-12 Interaction
STAI: State-Trait Anxiety Inventory
USA: United States of America
